# Comparing the OurRelationship Program and Bibliotherapy for Parents Experiencing Couple Distress: A Randomized Pilot

**DOI:** 10.1111/jmft.12762

**Published:** 2025-01-07

**Authors:** Tea L. Trillingsgaard, Frederik Godt Hansen, Hanne Nørr Fentz

**Affiliations:** ^1^ Department of Psychology and Behavioural Sciences, School of Business and Social Sciences Aarhus University Aarhus C Denmark; ^2^ Department of Political Science and Public Management University of Southern Denmark Odense M Denmark

**Keywords:** bibliotherapy, couple conflict, feasibility, online intervention, OurRelationship, self‐help

## Abstract

The OurRelationship Program is an 8‐h web‐based program for distressed couples adapted from Integrative Behavioral Couple Therapy. In this pilot randomized controlled trial, we aimed to replicate results from the founders and test feasibility criteria for program implementation across cultures. We randomized 39 Danish, opposite‐sex, distressed, parenting couples to either the OurRelationship program or self‐guided bibliotherapy (a self‐help book with a reading guide). Results showed significant within‐group changes in couple and individual functioning; for the OurRelationship, small‐to‐large effect sizes (*d* = 0.46–1.35 for relationship functioning, *d* = 0.29–0.80 for individual functioning), and for Bibliotherapy, small‐to‐moderate changes (*d* = 0.49–0.75 for relationship functioning, *d* = 0.28–0.45 for individual functioning). Differences between conditions were insignificant, except for moderate differences in reductions of communication conflict (*d* = 0.79) and relationship negatives (*d* = 0.43) in favor of OurRelationship. While feasibility results were positive, a sustainable implementation requires a resourceful organization and large‐scale delivery.

## 1 Introduction

High levels of couple conflict and distress take their toll on happiness, well‐being, and health for adults and children alike (Robles et al. 2014; Slatcher and Selcuk 2017). Among adults, couple distress increases the risk of psychological and physical partner violence (Spencer, Stith, and Cafferky 2022; Stith et al. 2008). For children, exposure to high levels of conflict and aggression among parents is associated with poor emotional, behavioral, and cognitive functioning in childhood and beyond (e.g., Cummings and Davies 2002). In the context of Denmark, recent empirical data show a relatively high prevalence with one out of five couples suffering from relationship distress (Trillingsgaard, Sørensen, and Fentz 2019) and 18.5% of pregnant couples reported past‐year physical or psychological partner aggression (Trillingsgaard et al. 2019). Also, about one in two Danish marriages end in divorce (Eurostat 2021).

The efficacy of face‐to‐face couple therapy in the treatment of couple conflict and distress is well‐established (Roddy et al. 2020). Yet, in Denmark, as elsewhere, there is a wide gap between the number of couples who need help and the number of couples who receive any couple therapy. Less than one in ten Danish couples who are currently distressed have sought any type of help as a couple (Trillingsgaard, Sørensen, and Fentz 2019). Also, according to the Danish public law on social service, Danish municipalities are obligated to provide a coherent system of preventive and targeted services to youth and to promote free‐of‐cost family‐oriented counseling to parents who seek help to alleviate family issues. Yet, only 37% of municipalities report having some type of joint counseling available to parenting couples (Politiken 2019).

From the literature, we know that couples would like to use online interventions in their help‐seeking (Doss et al. 2017; Georgia Salivar et al. 2020; Trillingsgaard, Sørensen, and Fentz 2019). An easy‐access and efficacious online program for distressed couples could break down some of the current barriers to seeking help and could fill an important gap in building a coherent system of public family intervention.

To increase access and address barriers to help‐seeking, the OurRelationship program (OR), an 8‐h, web‐based, primarily self‐help program for couples, was developed in the United States (Doss et al. 2013). The OR program was founded by professors Brian Doss and Andrew Christensen as an online self‐help version of Integrative Behavioral Couple Therapy (IBCT; Christensen, Doss, and Jacobson 2020; Doss et al. 2013). IBCT is a relatively recent approach to couple therapy that integrates different treatment strategies within a consistent behavioral theoretical framework (Christensen, Doss, and Jacobson 2020). Within the IBCT framework (2020), a couples’ core issue is formulated in terms of between‐partner differences (e.g., in personality, in preferences), emotional sensitivities (e.g., sense of worth, fear of loss), external stressors (e.g., job, contextual discrimination), and patterns of communication (e.g., escalation, avoidance). In IBCT, the dual intervention goals are fostering emotional acceptance of each other and facilitating behavioral change around mutual issues. The OR program assists the couple in formulating a shared understanding of their core issue and structures the couple's communication in a way that facilitates processes emotional of acceptance and change (Doss et al. 2013).

The founders of OR demonstrated strikingly positive and long‐term results of the program on both couple and individual functioning within a nationally representative sample of distressed American couples (Doss et al. 2016, 2019) and later in two unrelated samples of low‐income couples in the United States (Doss et al. 2020; Hatch et al. 2022).^1^ With numerous published reports from across these three different randomized controlled trials, the OR currently holds the strongest empirical support among available web‐based relationship programs (e.g., Doss et al. 2020; Mitchell et al. 2023; Roddy, Rhoades, and Doss 2020; E. G. Georgia Salivar et al. 2018, 2020). Implementing such a program in a Danish context could be a way to lower barriers and reach more distressed Danish couples.

In defining couple distress, we align with Christensen, Doss and Jacobson (2020) who understand a distressed relationship as one in which the emotional reactions to, and cognitive appraisal of, the partners’ interactions hold (a) a surplus of negativity or (b) a paucity of positivity or c) both a surplus of negativity and a paucity of positivity. Most distressed couples report both a surplus of negativity and a paucity of positivity, such as when a couple experiences that much too often their interactions lead to anger or resentment *and* they have lost their sense of joy, warmth, reward, or connection. However, for some couples, only one of these dimensions constitutes their main sense of distress, for example, a couple who is busy taking care of their careers while raising young children may primarily feel that they have lost a sense of connection even if they experience little anger or overt conflict in their interactions.

In recent years, technology‐based treatments have gained significant territory within treatment research in relation to most common mental health problems, with meta‐analyses demonstrating that these treatments produce outcomes comparable to their face‐to‐face counterparts (e.g., Carlbring et al. 2018; Hedman‐Lagerlöf et al. 2023). However, the population impact from these innovations ultimately depends on the relationship between value and cost as perceived by clients, practitioners, and real‐world organizations. The Center for Family Development, the Danish practice partner within this project, is a not‐for‐profit organization with an interest in disseminating empirically supported couple interventions to public (e.g., municipalities) and private (e.g., insurance companies) organizations. The organization made its staff and management available for working with the OR and evaluate the feasibility of OR implementation for this study. As reviewed below, previous RCTs have compared the OR to either a waitlist, an alternative online program, or both. From a practice point of view, an essential question is if OR can add value beyond the existing non‐tech alternatives. Among these alternatives, research‐based self‐help literature (bibliotherapy) is the most available low‐cost type of self‐help that clinical organizations could recommend to couples today. In Denmark, bibliotherapy—with non‐religious content—is widely accepted and used within mental health care due to its ease of dissemination. Also, bibliotherapy has been used in previous research on brief couple interventions (e.g., Babcock et al. 2013; Barton et al. 2018; Halford, Sanders, and Behrens 2001), making it a suitable active control condition, which also allows a stronger test of the OR as compared to a waitlist condition.

### 1.1 Aims

The overall research questions of this study were to what extent the OR program is (1) effective and (2) feasible in a different cultural context (i.e., in a Danish clinical setting). More specifically, the current study conducted a pilot study for question 1 and a full test of question 2. The value of a subsequent full‐scale randomized controlled trial on the effectiveness of the OR program depends firstly on the feasibility of program implementation across contexts and secondly on the success of the piloted study methods for determining effectiveness. Thus, this pilot study takes the first step in the direction of testing whether the OR is a feasible intervention that may be able to reach more distressed couples effectively across contexts. A greater understanding of the feasibility can inform potential future implementations of the OR. In this study, we addressed feasibility in terms of completion, coaching, costs, price expectancy, and technical and organizational setup.

### 1.2 Empirical Support

Prior RCTs by program founders have used waitlist designs to investigate the efficacy of the OR in a nationwide sample of 300 distressed couples (Doss et al. 2016, 2019), which is used as the main benchmark for the current study. Doss et al. (2016) found a medium‐sized effect on relationship satisfaction (between‐groups *d* = 0.69). The within‐group effect for the OR group on relationship satisfaction was large (*d* = 0.96) while the waitlist control group experienced a small increase (*d* = 0.27). In terms of individual functioning, Doss et al. (2016) found small to medium‐sized effects for the OR group, including depressive (*d* = 0.40) and anxious symptoms (*d* = 0.57), work functioning (*d* = 0.23), perceived health (*d* = 0.32), and quality of life (*d* =0.67). Couples in the waitlist control condition experienced small improvements in depressive symptoms (fewer symptoms), perceived health, and work functioning (*d* = 0.03‐.12), while they increased more in quality of life (*d* = 0.51) and reduced anxious symptoms (*d* = 0.38)^2^. This resulted in generally small‐sized between‐group effects although with larger effects for participants with initial difficulties in areas of individual functioning (Doss et al. 2016). Initial gains on both relationship and individual outcomes were maintained after 12 months (Doss et al. 2019). Evidence from recent RCTs with low‐income couples has also demonstrated that the OR produced medium‐sized improvements in relationship functioning and small‐sized increases in individual well‐being compared to waitlist control groups (Doss et al. 2020; Hatch et al. 2022; Roddy et al. 2021; Roddy, Rhoades, and Doss 2020). However, in the following section, we use the results from the nationwide RCT (Doss et al. 2016, 2019) as a benchmark for our expectations of effect sizes for OR as we did not have a particular aim of reaching low‐income couples.

While prior empirical research on bibliotherapy is scarce, one RCT contrasting self‐PREP with bibliotherapy indicated that low‐risk couples receiving bibliotherapy experienced a small improvement in relationship satisfaction (Halford, Sanders, and Behrens 2001). Also, in a different randomized clinical trial, Babcock et al. (2013) found that couples randomly assigned to bibliotherapy experienced a small increase in marital satisfaction from pre‐ to post‐assessment. However, Barton et al. (2018) reported that low‐income couples receiving bibliotherapy experienced a decline in couple functioning.

### 1.3 Expectations

We expect that the findings from the original RCT (Doss et al. 2016, 2019) on the efficacy of the OR on both couple and individual functioning are replicable across settings (from university to clinical setting) and cultures (from the United States to Denmark). Table 1 provides an overview of the expected effect sizes. Previous RCTs on the efficacy of the OR (as detailed above in “Empirical support”) have directly informed the expected within‐group effect sizes for the OR. The primary comparison was between‐group effects, but as the current study was a pilot study with limited statistical power, we also aimed to test within‐group effects with the expectation that our results would point in the same direction as previous evidence on the efficacy of OR from the original studies (Doss et al. 2016, 2019). Thus, we expect to replicate effect sizes. While we may not reach statistical significance at conventional levels (i.e., *p* < 0.05) due to the smaller sample size, it is valuable to focus on effect sizes, as they are not influenced by sample size.

**Table 1:** Overview of expected effect sizes (Cohen's *d*).

Outcome	Within‐group effect sizes for OR	Within‐group effect sizes for BIB	Between‐group effect sizes
Relationship functioning	Medium‐large	Small	Small‐medium
Individual functioning	Small‐medium	Small	Small

In terms of feasibility criteria, we expect that indicators of success regarding program delivery (i.e., client satisfaction, completion rate, and efficiency in delivery) and implementation of the program (i.e., technical and organizational setup) will be replicable in a Danish real‐world clinical setting (see able S2 in the Supporting Information S1 for full details on pre‐defined feasibility criteria). The interpretation and report of feasibility data were conducted in collaboration with the Center for Family Development.

None of the initial findings from the founders of the OR have been replicated by independent research groups and the program was not previously transferred to outside of the United States. If these results are to be replicable from an independent research group and across cultures, this will solidify the evidence base for the program and speak to its potential for distressed couples in both Denmark and beyond.

This study was designed to balance rigor with practical relevance, and it integrates both efficacy and effectiveness research elements. Efficacy aspects ensured rigor by recruiting participants through strict inclusion criteria, employing controlled random allocation, and assessing the same outcomes as the original efficacy trial. The intervention was delivered by highly trained staff with closely monitored protocol adherence, maintaining high fidelity. Effectiveness aspects emphasized real‐world applicability by testing the intervention across diverse cultural contexts and focusing on usability in a specific clinical setting. The comparison condition, “existing options for self‐help,” reflects a practical, real‐world alternative. Because the intervention and comparison conditions differ by more than one therapeutic factor, we apply the term effectiveness for the current study.

## 2 Methods

### 2.1 Participants

A total of 39 opposite‐sex couples (78 individuals) participated in the study. Table 2 provides an overview of the background characteristics of study participants. Our sample reflected the Danish population well on rates of schooling, employment, and marriage while couples with couple therapy experience (7% in the population) and a master's degree (15% in the population) were somewhat overrepresented as compared to Danish couples in the population (Trillingsgaard, Sørensen, and Fentz 2019).

**Table 2:** Demographic characteristics of participants (*n* = 78).

Demographic characteristic	All		Women		Men	
	*M*/%	SD	*M*/%	SD	*M*/%	SD
Age (mean years)	38.8	8.2	38	9.2	39.5	7.3
Relationship length (mean years)	12.8	7.8				
Married (%)	69					
Completed high school (%)	77		90		64	
Further education (%)						
No further education (%)	6		3		10	
Short further education (%)	28		18		39	
Bachelor's degree (%)	31		38		23	
Master's degree or higher (%)	35		41		28	
Previous experience with couple therapy (%)	32					
Currently employed (%)	87		82		92	
Age of youngest child (years)	5.6	4.6				

### 2.2 Procedure

Participants were recruited through a combination of organic searches and paid advertising on Google. The recruitment began on August 26, 2020, with the launch of the Danish OR website (www.OurRelationship.dk). With a cost of DKK 30,000 for *Ad Spend*, which represents the portion of the marketing budget dedicated to the specific campaign on Google, the link for the study website was displayed as one of the top hits in response to Google searches (search terms like “relationship issues” and “fighting with my partner”) and as advertisements within Google mail (Gmail) across 4 months.

During the study period, a total of 2600 users had entered the website, 92 couples had filled in the screener, and of these, 28 were eligible and included in the study. An additional Ad Spend of DKK10,000 ( ~ US$ 1450) on paid advertisements occurred to extend advertisement across two additional months. Another 11 eligible couples were recruited. Most traffic on the website (90% of visitors) came from Google advertisements.

On the website, visitors were instructed to complete an online consent form before filling in a screening survey to determine eligibility for the study. Because we only translated the opposite‐sex program version, we included only opposite‐sex couples. The experience of couple distress was an inclusion criterion. To capture distress dimensions of both high negativity and low positivity, we used a dual inclusion criterion in which couples were included if they reported high conflict aggression (but excluded for severe violence, fear, or injury), low relationship satisfaction, or both high conflict aggression and low relationship satisfaction. More specifically, couples were included if a) one or both partners reported any conflicts that led to at least one act of psychological aggression (e.g., insults, name‐calling) or one act of physical aggression (e.g., shoved, pushed, grabbed) in the past year (but excluded if these acts led to injury or fear of safety) or/and b) one partner scored at least one standard deviation (indicating severe relationship distress) or both partners 0.5 standard deviations below the Danish population mean on relationship satisfaction (Couple Satisfaction Index, CSI, Funk and Rogge 2007). In addition, partners had to be at least 18 years old, living together for at least 6 months, have one or more child(ren) below the age of 18 living at home, and not in treatment with couple therapy or medications prescribed to treat a mental health condition (to reduce interference with treatment effect). Both partners had to be able to read and understand Danish, have access to the internet, and be willing to refrain from couple therapy within the period of the project. Couples were excluded if one or both partners reported conflicts leading to injury or fear of safety. Also, having an ongoing affair or being in the process of divorcing or breaking up led to exclusion. These exclusion criteria reflect that IBCT, and thereby also OR, is “for couples who have an established relationship and are committed to each other, even if ambivalently so” (Christensen, Doss, and Jacobson 2020, 75). Also, a general criterion for a couple being suitable for IBCT is that there is no physical danger such as if recent conflicts have led to injury or fear of physical harm. Previous research on IBCT has shown that couples often come to therapy with low levels of violence (e.g. escalating arguments) and that those couples can be treated (Simpson et al. 2008). Therefore, couples with conflict aggression were included but those whose conflict aggression resulted in injury or fear in the past 3 months were excluded. To reduce the risk of dangerous self‐harm, couples were also excluded if either partner reported moderate to severe levels of suicidal ideation ( > or = 7 on the Suicidal Behavioral Questionnaire‐Revised, Osman et al. 2001). If a couple was eligible for the study, each participant (partner) separately received a telephone call from the project staff (a student assistant). Each partner was provided with oral information on the project and an opportunity to ask questions. During the call, staff ensured that both partners were motivated, had the time available, and checked that inclusion criteria were fulfilled. A written consent form was e‐mailed to be filled in individually and returned before inclusion. Finally, included couples were invited to fill in the first questionnaire and then randomized for one of the two conditions.

### 2.3 Randomization and Interventions

For the randomization procedure, we used sequentially numbered, opaque, sealed envelopes (SNOSE; Doig and Simpson 2005) that were shuffled and contained a sheet with information on either belonging to Treatment A (OR) or Treatment B (bibliotherapy). Upon inclusion, a research assistant added the couples’ ID to the next envelope in line, which was then opened. Consequently, 22 couples were randomized to the OR condition, and 17 couples were randomized to receive bibliotherapy. Results from *t*‐tests showed that the two treatment groups were balanced on background characteristics (*p* > 0.05) except for the proportion of married couples (results not shown). With a small sample and no other systematic differences, we assume that the difference in marriage was random.

#### 2.3.1 OR Condition

A copy of the OR (opposite‐sex version) was installed onto servers at Aarhus University, and all text materials including coach manuals and training materials were translated into Danish. Video clips and sound pieces were subtitled. Couples completed the program at their homes, which included about 6–8 h of online activities over a period of 6–8 weeks. The content consists of three online modules: Observe, Understand, and Respond. In the Observe phase, each partner identifies a core problem in the relationship on which they want to work during the program. In the Understand phase, each partner develops a comprehensive understanding of the core problem(s) from both their own and their partner's perspective by means of a DEEP analysis (i.e., Differences, External Stress, Emotions, Patterns of Communication). In the Respond phase, each partner works with the themes of both acceptance and change (take action in relation to the core problem). During each module, the partners complete individual activities first and then meet to have a joint conversation that follows a structured format and is guided by the program. After each joint conversation, the couple receives a tightly scripted coach call scheduled to last 20 min. In addition, a first coach call is scheduled before couples start the program. For this study, coaches included three experienced clinical psychologists and two interns in the Center for Family Development who had a bachelor's degree in psychology.

#### 2.3.2 Bibliotherapy Condition

The active control group was designed to mirror the benefit that couples are likely to get from engaging in the existing self‐help option in Denmark: using well‐chosen, solid literature. In replacement of the self‐help book recommended as the adjunct to IBCT (Christensen, Doss, and Jacobson 2014), which was not available for a Danish audience, we selected a high‐quality book currently in use by our practice partner. The book “Take Care of Your Relationship When Love Is Here to Stay” [Danish: “Pas på parforholdet, når kærligheden er kommet for at blive”] by Mattias Stølen Due (2016) is a science‐based, light‐density guide to couples. The book refers to research‐based knowledge of prosocial behaviors and attitudes in couple relationships from a variety of sources (e.g., Christensen 2010; Cordova 2009; Gottman and Silver 1999; Halford and Markman 1997; Jacobson and Christensen 1998; Lyubomirsky 2007). Each of the eight chapters focuses on one behavior or attitude that each can do to enhance the relationship (e.g., be a friend, aim to understand, address issues, pursue gratitude) and includes a number of questions for reflection and discussion. Couples in the active control group received two copies of this book (or an e‐book upon request) and a two‐page readers’ manual providing guidelines on how to coordinate reading with your partner and schedule four 30–60‐min couple conversations. For these conversations, couples were instructed to choose two reflection questions from the book. Couples in the active control group worked on their own, and to reflect how bibliotherapy is used in practice, couples in this condition received no coach calls.

### 2.4 Measures

Both experimental groups completed surveys at baseline (inclusion), at post (about 8 weeks after inclusion), and at 3‐month follow‐up. All measures were translated from English to Danish by two of the authors, each making an independent translation of each measure. This was followed by a consensus discussion between the authors and consultation with the scale developer in case of disagreement. Besides the measures of intimacy and perceived stress, all outcome measures used to pilot test the effectiveness of the OR program were based on their previous use in the original studies on OR conducted in a US setting (Doss et al. 2016; Doss et al. 2020).

#### 2.4.1 Relationship Satisfaction

Global relationship satisfaction was measured by the four‐item version of the Couple Satisfaction Index (CSI‐4; Funk and Rogge 2007), with example items including “In general, how satisfied are you with your relationship?”. This item was scored on a 0–6 scale, while the other three items ranged from 0 to 5. Sum scores ranged from 3 to 18 (*M* = 9.64; SD = 3.84) at baseline, with higher scores representing greater satisfaction. Internal consistency for the CSI‐4 at baseline (Cronbach's *α* = 0.90) and follow‐up (Cronbach's *α* = 0.95) was excellent.

#### 2.4.2 Intimacy

A four‐item scale on psychological intimacy (Debrot et al. 2013) was used in the present study with example items such as “I feel close to my partner”. All items were scored on a 5‐point scale (0 = *does not apply*; 4 = *apply very strongly*). Baseline sum scores ranged from 0 to 15 (*M* = 7.71; SD = 3.40) and Cronbach's *α* was = 0.87 at baseline and = 0.90 at follow‐up.

#### 2.4.3 Relationship Confidence

In this study, relationship confidence was measured with two items from the Confidence Scale (Rhoades, Stanley, and Markman 2009), *“*I believe we can handle whatever conflicts will arise in the future” and “I feel good about our prospects to make this relationship work for a lifetime.” Both items were rated on a 7‐point Likert scale (1 = *strongly disagree*; 7 = *strongly agree*), and sum scores ranged from 2 to 14 (*M* = 9.23; SD = 2.77) at baseline. In the present sample, Cronbach's *α* = 0.83 (at baseline) and = 0.89 (at follow‐up).

#### 2.4.4 Positive and Negative Relationship Quality

We used the Positive and Negative Relationship Quality eight‐item self‐report scale (Fincham and Rogge 2010). Four items were directed at relationship positives (e.g., *“*My relationship is enjoyable”), while the remaining four items asked about negative dimensions of relationship quality (e.g., “My relationship is miserable”). All items were scored on a 7‐point Likert scale ranging from “not at” to “extremely.” Baseline sum scores on relationship positives ranged from 1 to 21 (*M* = 13.69; SD = 4.81), while sum scores ranged from 0 to 22 (*M* = 8.59; SD = 5.35) on relationship negatives. Cronbach's *α* for positives was 0.91 at baseline and 0.93 at follow‐up, while Cronbach's *α* for negatives was 0.89 at baseline and 0.90 at follow‐up.

#### 2.4.5 Communication Conflict

Negative communication was measured using a seven‐item measure developed for the Administration for Children and Families Supporting Healthy Marriage Initiative. For instance, participants rated how often “small issues suddenly became big arguments” over the past 2 weeks on a scale from 1 (“never”) to 4 (“often”). Baseline sum scores ranged from 8 to 27 (*M* = 18.65; SD = 4.94). Cronbach's *α* in the present sample was 0.87 at baseline and 0.93 at follow‐up.

#### 2.4.6 Depressive Symptoms

Depression was measured using the Major Depression Inventory (MDI; Olsen et al. 2003) by answering items such as “How much of the time have you felt low in spirits or sad?”. Response categories ranged from “all the time” (1) to “never” (6). Baseline sum scores ranged from 1 to 36 (*M* = 15.55; SD = 9.36), with higher scores indicating greater symptoms of depression. Cronbach's *α* in the present study was 0.89 at baseline and 0.91 at follow‐up.

#### 2.4.7 Quality of Life

One item from the Quality of Life—Brief developed by the World Health Organization (WHOQOL Group 1998) was used to measure quality of life. Participants were asked to answer the following questions on a 5‐point Likert scale ranging from “very poor” to “very good”: “How would you rate your quality of life?” (*M* = 3.47; SD = 0.86).

#### 2.4.8 Perceived Health

A one‐item question, “How satisfied are you with your health?” (WHOQOL Group 1998), was used to assess perceived health on a 5‐point scale ranging from “very dissatisfied” to “very satisfied” (*M* = 3.29; SD = 1.11).

#### 2.4.9 Perceived Stress

Four items from the Perceived Stress Scale (Cohen, Kamarck, and Mermelstein 1983) were used to determine participants’ level of perceived stress. For instance, participants answered how often (in the past 2 weeks) they have felt that they were unable to control the important things in their life?, with response categories ranging from “never” (1) to “very often” (5). Baseline sum scores ranged from 0 to 12 (*M* = 6.65; SD = 3.02), and internal consistency was excellent (baseline Cronbach's *α* = 0.85 and follow‐up Cronbach's *α* = 0.84).

#### 2.4.10 Work Functioning

The item “Please rate your ability to function at work. If you do not work outside the home, please rate your ability to complete household tasks” was used to survey participants’ work functioning. Responses were scored from 1 (“poor”) to 5 (“excellent”) (*M* = 3.58; SD = 0.95).

#### 2.4.11 Couples' Evaluation of the Intervention

The eight‐item Client Evaluation of Services Questionnaire (Nguyen, Attkisson, and Stegner 1983) was administered after the intervention and estimated couples’ satisfaction with the services provided. An item example is: “How would you rate the quality of service you received?” on a 4‐point scale ranging from “excellent” (1) to “bad” (4). Sum scores ranged from 9 to 32 (*M* = 24.72; SD = 5.20). Cronbach's *α* was 0.95.

#### 2.4.12 Price Expectancy

The item “If you were to pay for an online program with coaching, as the OR, which price would you consider reasonable?” was used to survey how much participants would be willing to pay for the OR with coach support. Response categories ranged from 0 (0 DKK) to 6 (4000 DKK).

#### 2.4.13 Completion

We followed definitions of completion used in the benchmark RCT (Doss et al. 2016) which were the completion of the Observe and the Understand phases including coach calls. We defined completion of bibliotherapy as reading at least half of the book and engaging in two or more hours of couple conversations related to themes in the book. In rough estimates, completion of the OR required at least 6 h, while completion of the bibliotherapy required at least 4 h.

#### 2.4.14 Coaching

Time spent on coaching was measured as the length of the coach calls in minutes. Administration time includes rescheduled calls and e‐mail communication. While the US version of the OR adopts system‐generated email reminders before each call, our coaches sent encrypted emails manually, which was necessary to comply with General Data Protection Regulation (GDPR) requirements in Europe.

### 2.5 Organizational Feasibility: Technical Demands, Organizational Demands, and Costs

A Danish Feasibility Report was authored by a key staff member and the executive manager of the Center for Family Development (Center for Family Development 2021). This report made use of experiences working with the OR program from coaches, clients, other staff members, and the management. Authors had access to all findings included in this paper as well as nine qualitative client interviews conducted and summed by a research assistant on client experiences. For the purpose of this article, we refer to the Danish Feasibility Report as a source for evaluating organizational feasibility.

### 2.6 Missing Data

**Figure 1:**
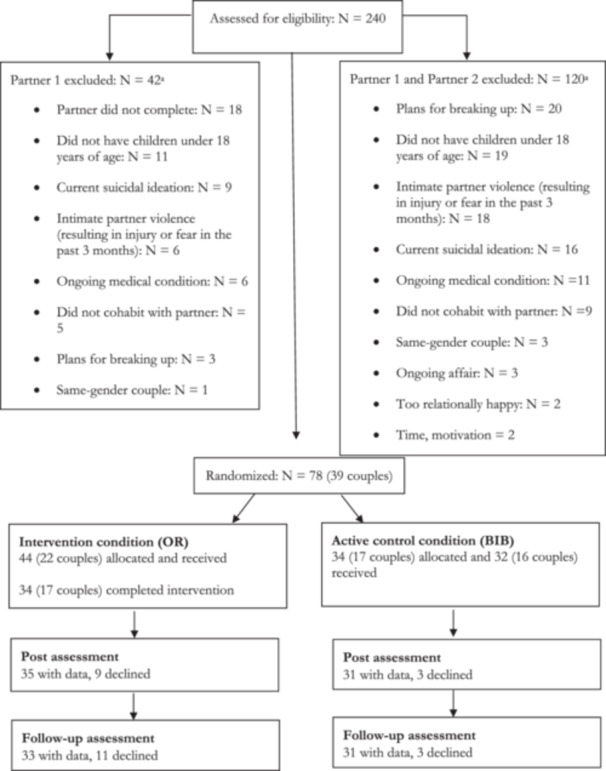
Flowchart. *Note:* ^a^Numbers do not sum to 42 participants or 120 couples because some individuals or couples were ineligible for multiple reasons.

Dropout from the study caused some missing data over time. Across the two conditions, post‐assessment data were missing for 15% (66/78 completed the survey and had valid data on the outcome measures), while 18% was missing at follow‐up (64/78 completed the survey). This amount of missing data is comparable to previous studies on the OR (Doss et al. 2016). All available data were included in the analyses (see Figure 1).

## 3 Analyses

To obtain estimates of change, we analyzed all outcomes with multilevel models (mixed‐effects models) as the data have a nested structure with time nested in individuals and individuals nested within couples. Even though the small sample size imposes some limitations on how strong inferences we can make from the multilevel model, we chose this model due to the nested data structure. Also, we aimed for a simple multilevel model (e.g., without adding other explanatory factors) due to the smaller sample size. Based on visual inspections of outcome means over time, we specifically chose a two‐level random intercept model with time (level 1) nested within individuals (level 2). We also fitted an unconditional model for each outcome to inspect the random effects concerning the intercept (see Table 3). The variance of the intercepts indicated the need for a multilevel model with random intercepts. Time could take three values (pre, post, and follow‐up) and was entered as the only predictor at level 1. To handle missing data across time points, we employed Full Information Maximum Likelihood to estimate the parameters, which used all available data from each respondent even if data is missing at some time points.

**Table 3:** Multilevel results for relationship outcomes.

	Relationship satisfaction					Intimacy						Communication conflict					
	Null		Level 1		Level 2		Null		Level 1		Level 2		Null		Level 1		Level 2	
Variable	*b*	*SE*	*b*	*SE*	*b*	*SE*	*b*	*SE*	*b*	*SE*	*b*	*SE*	*b*	*SE*	*b*	*SE*	*b*	*SE*
*Fixed effects (int. period)*																		
Change			2.96***	0.45	2.56***	0.68			2.63***	0.44	2.50***	0.66			4.95***	0.66	−2.78**	−0.93
Condition					0.41	1.06					0.31	1.03					2.36	1.45
Change* condition					0.75	0.90					0.26	0.88					−4.01***	1.14
Intercept	11.44***	0.54	9.64***	0.53	9.41***	0.79	9.17***	0.45	7.71***	0.49	7.53***	0.85	15.90***	0.68	18.65***	0.73	17.32***	1.15
*Fixed effects (FU‐period)*																		
Change			0.14	0.45	0.21	0.39			−0.34	0.28	−0.21	0.39			0.75	0.71	−0.92	0.93
Condition					1.15	1.16					0.57	1.01					−1.65	1.56
Change* condition					−0.13	0.60					−0.24	0.56					−0.34	1.40
Intercept	11.44***	0.54	12.60***	0.57	11.98***	0.99	9.17***	0.45	10.34***	0.49	10.04***	0.82	15.90***	0.68	13.70***	0.77	14.54***	1.28
*Random effects*																		
Variance (intercept)	10.88	2.39	11.10	2.20	10.91	2.11	6.55	1.60	6.75	1.53	6.69	1.49	12.91	3.84	14.50	3.27	15.38	3.43
Residual variance	7.39	1.09	4.40	0.61	4.37	0.61	6.15	0.96	4.16	0.65	4.16	0.66	19.46	3.10	12.36	1.80	10.75	1.78
*N*	208		208		208		207		207		207		207		207		207	

*Note:* Unstandardized coefficients from multilevel models with clustered standard errors (couple level). Clusters = 39 couples. Change is a time variable coded as pre = 0; post = 1; FU = 2 for the intervention period and coded as post = 0; FU = 1; pre = 2 for the follow‐up period. Condition is coded as 0 = BIB and 1 = OR.

* *p* < 0.05

** *p* < 0.01

*** *p* < 0.001.

To capture change during the intervention period (pre‐to‐post change), time was centered on pre‐assessment. Post‐assessment was coded as the base level to capture the maintenance of effects during the follow‐up period (post‐to‐follow‐up change). The intervention condition was entered as a level‐2 predictor of both the level‐1 intercept and slope. This enables us to determine whether the change over time is different across treatment conditions. To correct for the case that individuals within couples share unobserved characteristics (individuals clustered in couples), all models included cluster‐robust standard errors at the couple level (McNeish, Stapleton, and Silverman 2017).

Within‐group effect sizes (Cohen's *d*) were calculated as the pre‐to‐post change for each intervention condition divided by the pooled pre‐ and posttreatment standard deviation. Due to the small sample size of this study, changes over time for each intervention condition are presented to better explore the effectiveness of the OR, even though it is important to note these changes cannot be attributed solely to the intervention (but may be caused by the positive spill‐over of agreeing on signing up for a couple intervention, increasing hope for change, or completing questionnaires). Between‐groups Cohen's *d* was calculated by group differences in slopes at post‐assessment divided by the pooled pre‐ and posttreatment standard deviation.

## 4 Results

### 4.1 Changes in Relationship Outcomes

**Figure 2:**
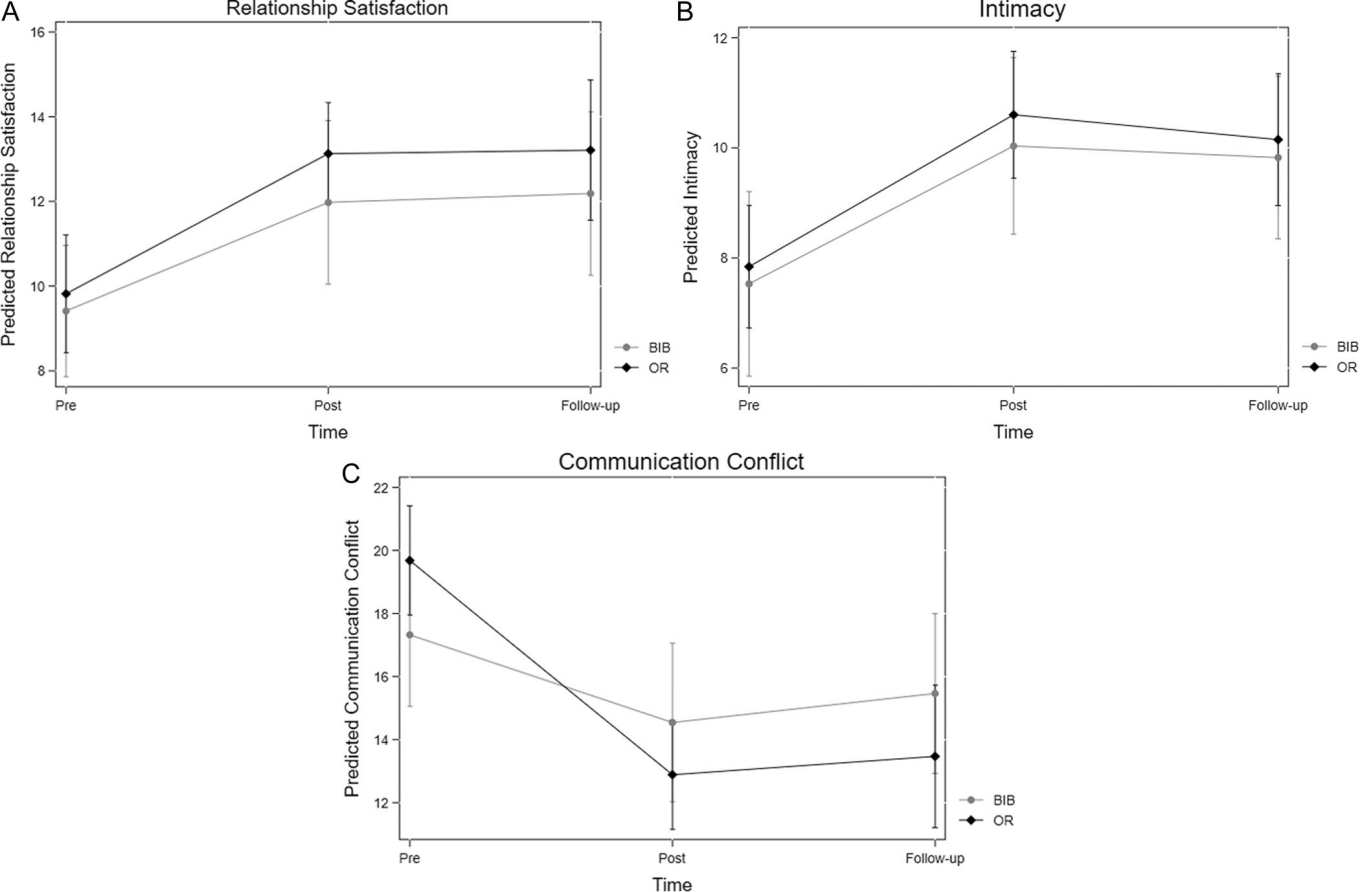
Multilevel results: Relationship outcomes over time note. The graphs are based on the models including a time‐by‐treatment interaction in Table 3. The graphs show the marginal effects of each treatment condition (BIB or OR) over the intervention period and follow‐up, respectively. BIB, bibliotherapy; OR, OurRelationship.

Based on previous work on the OR (Doss et al. 2016), we focused at first on relationship satisfaction, intimacy, and communication conflict as the main relationship outcomes. Thus, regression results from multilevel models on these outcomes are provided in Table 3, while Figure 2 presents within‐group changes over time for the two conditions separately.

Overall, results revealed that participants across conditions experienced a statistically significant positive change in all three relationship outcomes over the intervention period from pre‐ to post‐assessment (see the row for “change” in level 1 models in Table 3). Examining first within‐group changes, individuals receiving the OR experienced large improvements in relationship satisfaction (*b* = 3.31, *p* < 0.001, *d* = 0.87), intimacy (*b* = 2.76, *p* < 0.001, *d* = 0.82), and communication conflict (*b* = −6.79, *p* < 0.001, *d* = 1.35). Individuals within the bibliotherapy group experienced medium‐sized improvement in relationship satisfaction (*b* = 2.56, *p* < 0.001, *d* = 0.67), intimacy (*b* = 2.50, *p* < 0.001, *d* = 0.75), and communication conflict (*b* = −2.78, *p* < 0.01, *d* = 0.56). In relation to between‐group changes, individuals receiving the OR experienced significantly greater reductions in communication conflict compared to the bibliotherapy condition, which were of a medium‐sized effect (*b* = −4.01, *p* < 0.001, *d* = 0.79).

**Figure 3:**
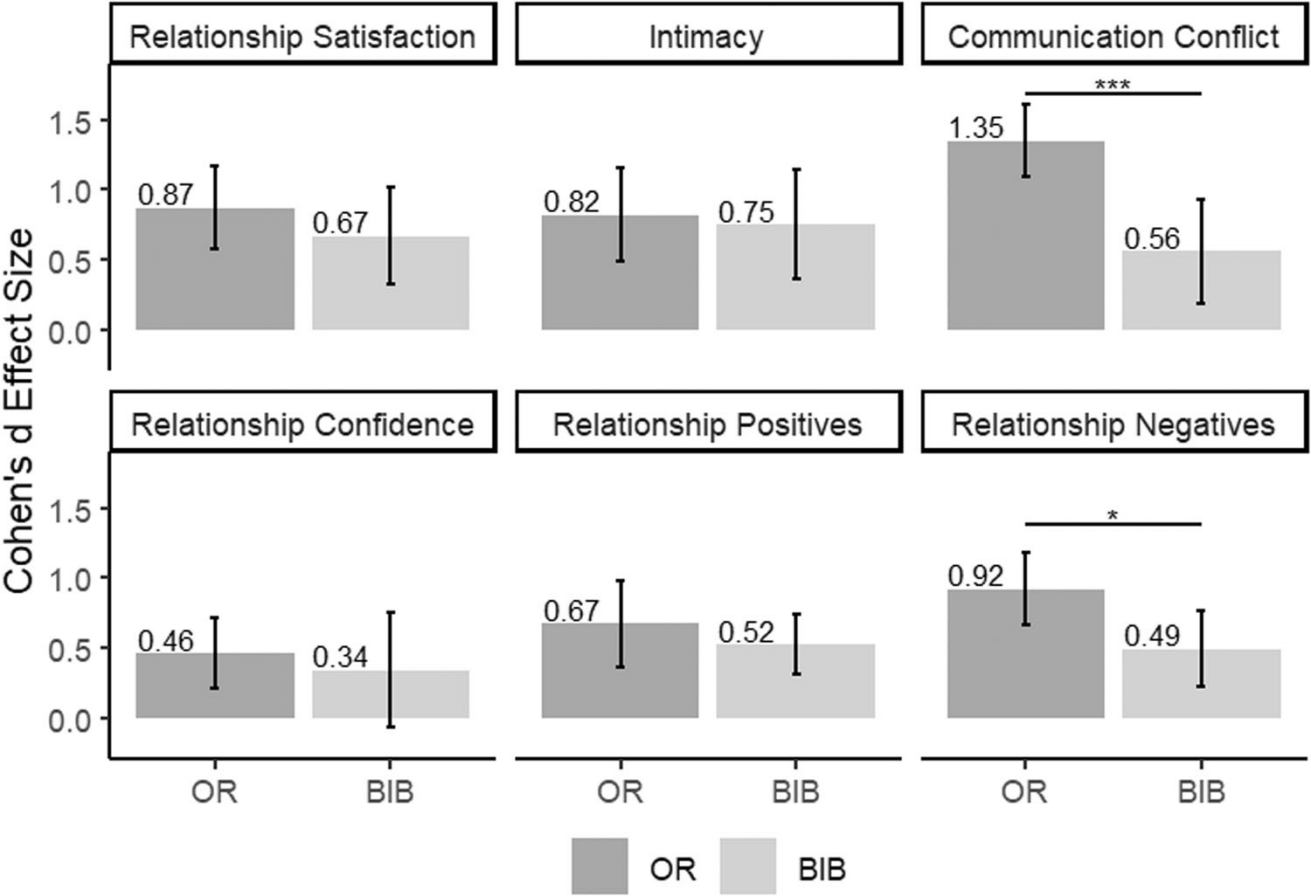
Within‐group effect sizes for relationship functioning. *Note:* The figure is based on multilevel models. BIB, bibliotherapy; OR, OurRelationship. *p < 0.05; **p < 0.01; ***p <0.001.

Figure 3 provides an overview of the within‐group effect sizes for both experimental groups across the full set of relationship functioning outcomes. As shown in Figure 3, in the OR, relationship confidence improved with an effect size approaching a medium effect (*d* = 0.46), and medium to large improvements were seen in relationship positives (*d* = 0.67) and relationship negatives (*d* = 0.92). Among these additional measures of relationship functioning, the only significant difference between the conditions was a larger reduction in relationship negatives (between‐groups *d* = 0.43, *p* < 0.05). Visual inspection shows other between‐group differences in the expected direction in the very small range (*d* = 0.07–0.20, *p‐*values = 0.41–0.77), but without a larger sample, we cannot rule out that these were caused by random error.

Across all relationship functioning outcomes, the initial gains were maintained in the 3 months following the program, as individuals within each group experienced no significant change—neither statistically nor practically—in any relationship outcome (*p*‐values = 0.35–0.83); see Figure 2 or the middle panel of Table 3 for results covering the follow‐up period.

### 4.2 Changes in Individual Outcomes

**Figure 4:**
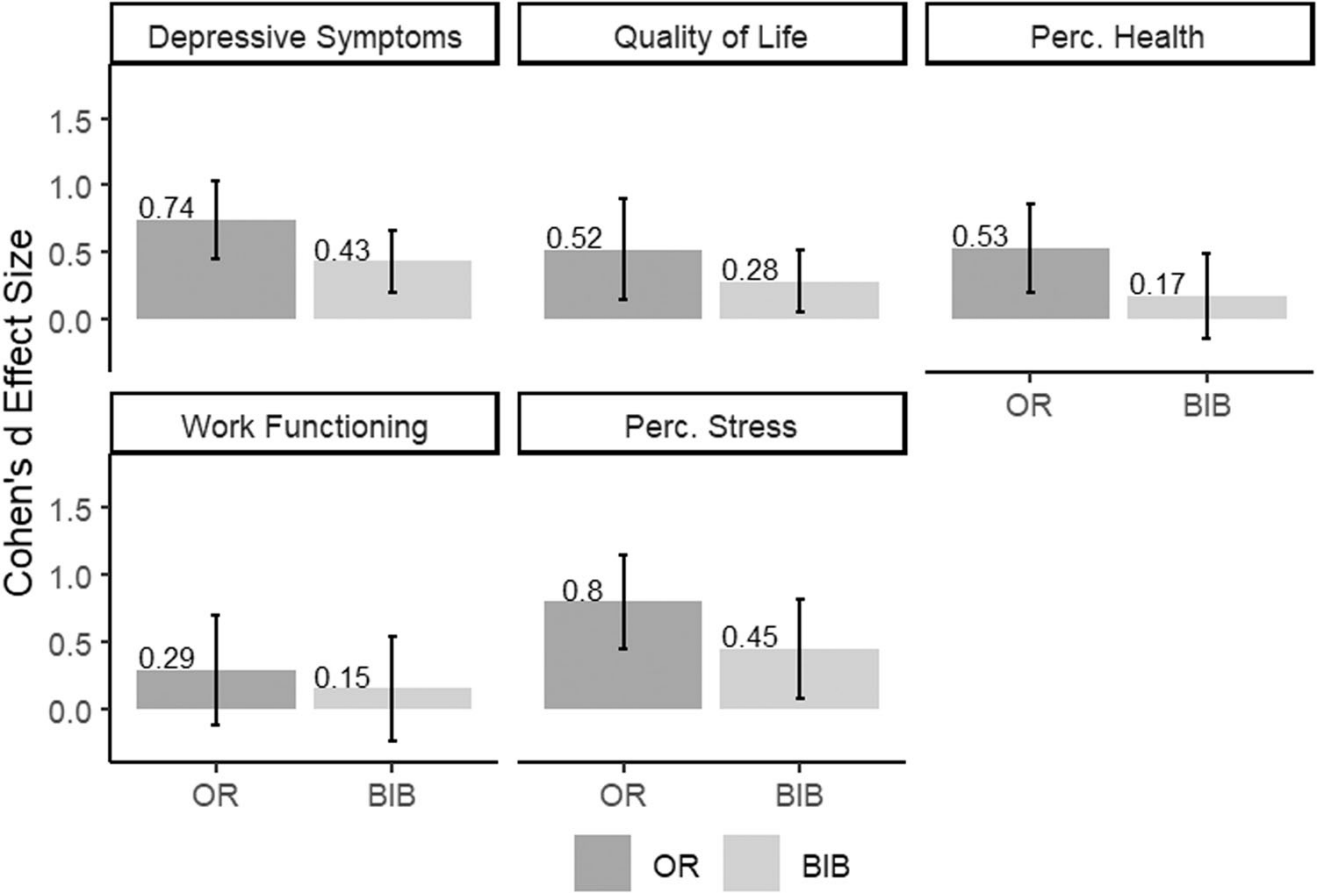
Within‐group Effect Sizes for Individual Functioning. *Note:* The figure is based on multilevel models. BIB, bibliotherapy; OR, OurRelationship. *p < 0.05; **p < 0.01; ***p < 0.001.

Effect sizes for individual outcomes are presented in Figure 4. Participants assigned to the OR condition experienced significant improvements (all *p*s < 0.01) of medium‐to‐large‐sized effects in depressive symptoms (*d* = 0.74), quality of life (*d* = 0.52), perceived health (*d* = 0.53), and perceived stress (*d* = 0.80). In the bibliotherapy condition, participants also improved but effect sizes were in the small range (*d* = 0.28 to 0.45), while changes in perceived health were very small and did not reach statistical significance (*d* = 0.17; *p* = 0.27). The effect size for work functioning was small (*d* < 0.30) and statistically insignificant for individuals in both conditions (*p* = 0.47 for bibliotherapy and *p* = 0.18 for OR).

The between‐group estimates were in the expected direction, but neither of them reached statistical significance in our small sample (*p*‐values = 0.11–0.66). To qualify the power analyses for future scaling of the experiment, we noted that the substantial between‐group effect sizes were of small magnitude for depressive symptoms (*d* = 0.31), quality of life (*d* = 0.24), perceived health (*d* = 0.36), and perceived stress (*d* = 0.35). Again, the effect size for work functioning was the smallest (between‐groups *d* = 0.14).

When looking at the follow‐up period, the initial gains in individual functioning were maintained as individuals in both conditions experienced neither significant improvements nor deteriorations both statistically and practically speaking (*p*‐values = 0.18–0.92).

### 4.3 Feasibility Results

#### 4.3.1 Couples’ Evaluation of the Intervention

Participants reported a generally high level of client satisfaction when evaluating the OR program with a mean score of 25.51 (SD = 5.58). A total of 86% reported they were satisfied or very satisfied with the services received, and 91% said they would recommend the service to a friend (40% answered, “Yes, I think so,” and 51% answered, “Yes, for sure”). Thus, the current client satisfaction rate was within the expected range of up to 0.3 standard deviations lower than the satisfaction score demonstrated in the benchmark RCT (*M* = 26.81, SD = 4.44) (Doss et al. 2016). In the Danish Feasibility Report, client interviews resulted in a call for a cultural adaption of the material used in this language version, for instance, using Danish actors in the videos and translating audio clips into Danish to achieve a better “Danish feel” of the program. These aspects of the Danish OR program may explain the somewhat lower evaluation score seen in this study compared to the benchmark RCT.

Participants in the bibliotherapy condition reported a mean score of 22.87 (SD = 4.42) when evaluating the intervention. A total of 68% reported they were satisfied or very satisfied with the services received, and 90% said they would recommend the service to a friend (61% answered, “Yes, I think so,” and 29% answered, “Yes, for sure”). The lower overall evaluation score of bibliotherapy compared to OR (22.87 vs. 25.51) and the lower number of participants reporting being satisfied or very satisfied (68% vs. 86%) indicate that couples receiving bibliotherapy generally were less satisfied with the services provided compared to the OR condition.

#### 4.3.2 Completion

Of the 22 couples randomly allocated to the OR condition, 17 couples (77%) completed the program. The completion rate was a little lower than expected (80%) and lower compared to the benchmark RCT (86%; Doss et al. 2016). Note, however, that in this small sample, 17 completions were only one completion short of reaching the expected completion rate.

Of the 17 couples randomly allocated to the Bibliotherapy condition, 87% (27 of the 31 individuals who answered post‐assessment in the bibliotherapy condition) reported that they had read at least 50% of the book (64% had read at least 75% of the book). Moreover, 84% (26 out of 31) reported that they had spent 2 or more hours on conversations with their partner about the themes in the book. A total of 77% (24/31) of individuals had both read at least 50% of the book *and* spent at least 2 h on conversations with their partner. In terms of engagement, all individuals reported having read at least 25% of the book.

#### 4.3.3 Coaching

Coaches spent an average of 27 min (range was 15–60 min) with couples on each call and 30 min on preparation and administration per call. Administration time included that couples on average had one rescheduled call, Junior staff spent 1 h in group‐based training and supervision per couple, and senior staff somewhat less. In total, coaches used about 5 h per couple. In the benchmark RCT (Doss et al. 2016), the reported total time used for all four coach calls is less than an hour (*M* = 51.32 min) plus time used on (an average of 5) scripted chat reminders. In comparison, the Danish coaches used significantly more time on preparation and administration of the calls than the US coaches. The bibliotherapy condition required no coach calls and involved less than 15 min of administration per couple for handling and shipping of the books.

#### 4.3.4 Technical and Organizational Setup

The Danish Feasibility Report (Center for Family Development 2021) is authored by the Center for Family Development and describes (among other parts of the evaluation) the technical and organizational demands related to implementing the Danish version of the OR. In this section, we utilize insights from the Report to emphasize findings of broad relevance, applicable in various contexts beyond the specificities of the Danish case and its local environment.

The first crucial task for disseminating OR in Europe involves building, hosting, and maintaining a program version on a digital platform within the European region (for instance, in Denmark). Owing to GDPR regulations in Europe, organizations are currently unable to request European individuals, such as clients or research participants, to sign up on the OurRelationship.com platform hosted in the United States, even if a relevant language‐specific program version was available. A nontrivial technical demand is that the OR program links individual users into couples so that data from individual users interact to shape the content. This feature is essential to a couple‐oriented program but requires customization of existing Learning Management Systems or a completely new built. Smaller updates are continuously implemented to the US version and larger updates are planned for every 4 years by the program founders. Thus, there is a day‐to‐day task of ensuring and updating local software functionality and there is a more complex task of making larger updates available and ensuring data security. Most clinical settings, including the Center for Family Development, are currently not staffed for the delivery of technology‐based interventions and will need at least one in‐house person with software knowledge in addition to an ongoing collaboration with a company specialized in digital solutions and data security. In addition to this, a staffed helpline is required to assist users with technical problems.

In addition to technical demands related to hosting the digital platform, three organizational demands are further described in the Danish Feasibility Report. First, attracting couples to a new and relatively unknown type of intervention requires a robust marketing strategy and outreach (e.g., through various channels, such as social media, e‐mail newsletters, the practice's website, and offline materials). Second, the cost‐effectiveness in the delivery of the OR depends on the availability of a less specialized workforce in the organization who take care of the contact to couples and provide the coach calls. Coaching can be conducted by less experienced therapists, such as psychology interns, psychology students, or skilled volunteers, if provided with structured training and close supervision. Thus, the implementation of the program requires a model for recruiting, training, and supervising a workforce of coaches. Third, a strategy to handle the potential organizational disruption is needed. The Danish Feasibility Report raises a concern that OR might divert couples from existing services. Since face‐to‐face therapy constitutes a significant part of these organizations’ core offerings, the emergence of alternative treatment options like OR could potentially reduce the demand for specialized personnel. At the same time, the Danish Feasibility Report underscores the possibility that OR, by reducing barriers such as cost, transportation difficulties, or social stigma, may attract new clients, collaborators, and funding sources. Moreover, implementing the OR allows for establishing nationwide service to couples. Various strategies are proposed to integrate OR into existing services while still using the potential of reaching and serving more couples. One such strategy is a blended format, where the online program is combined with face‐to‐face consultations (Christensen, Doss, and Jacobson 2020). Another is stepped care models which provide fluid transitions from online self‐help to more intensive face‐to‐face services for couples requiring additional support. A key conclusion from the Danish Feasibility Report is that the implementation of the OR would allow CFF to serve substantially more couples and that the center can meet the additional organizational demands of the OR.

#### 4.3.5 Price Expectancy

Asking participants allocated to the OR condition to estimate an acceptable price for the program (with a coach), 33% of participants reported that they would be willing to pay less than 134 euros (below DKK 1000) for the program, while 48% reported they would be willing to pay between 134 euros and 268 euros (DKK 1000–2000), which is about equivalent to the price of 1 session of couple therapy.

#### 4.3.6 Costs

**Figure 5:**
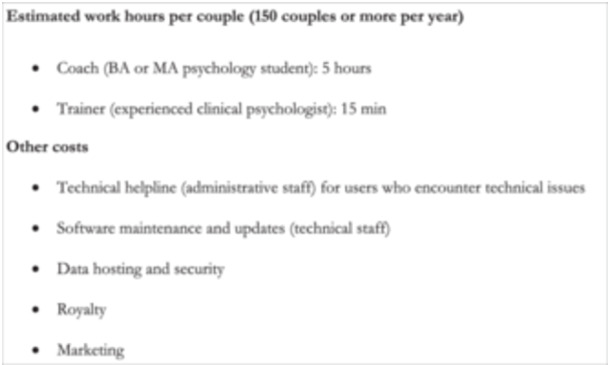
Items of expenditure for implementing the OR.

We refer to the Danish Feasibility Report for details on the estimated price model in which the OR can be self‐sustainable or return a small profit if offered for DKK 1000–2000 (~US$ 150–300), which was the maximum value interval that 48% of participants would be willing to pay. This price model conditions that the program is already culturally adapted and/or translated. The adaptations made for this study were minor and text only (e.g., replacing the word “car” with “transportation” as more Danes used public transportation), whereas major adaptations (e.g., clips with Danish actors) were not made. With the arrival of artificial intelligence, the workload involved in translation has diminished since the translation into Danish was conducted. The price model also conditions that an annual number of 150 couples or more are willing to pay for access with coaching and that larger (4‐year) updates to the platform are covered by external funding. If the OR reaches a higher number of couples, is offered with more automatization and less coaching, or if the program can attract public or charity funding, a lower price may be possible (at least in Denmark). We provide estimates in terms of work hours below (Figure 5).

In comparison, the price of two copies of the book including shipment is currently DKK 430 ( ~ US$ 63). Other relevant comparisons include both the cost and the effect of other brief couple interventions such as the Marriage Checkup (Cordova et al. 2014; Trillingsgaard et al. 2016) or PREP (Prevention and Relationship Enhancement Program; Stanley, Blumberg, and Markman 1999), but this is outside the scope of the current paper.

## 5 Discussion

The aim of this study was to pilot whether the effectiveness of the OR, solely demonstrated in the United States, could be replicated in another cross‐cultural context by an independent research group. Furthermore, we aimed to test the feasibility of implementing such an online resource in a clinical setting. The between‐group effect sizes were mostly nonsignificant but in the expected direction and size (small‐to‐medium) across all relationship functioning outcomes (e.g., relationship satisfaction and intimacy). The exceptions were communication conflict and relationship negatives, both of which were of moderate size and reached statistical significance (at the 5‐*%* level). The between‐group effect sizes for individual functioning (e.g., perceived stress and quality of life) were also nonsignificant, in the expected direction, and of the expected size. Even though within‐group changes cannot be interpreted as evidence of a treatment effect, the results from this pilot study showed that the OR fully met expectations regarding within‐group changes in relationship functioning (in the medium to large range) and outperformed the expectation regarding within‐group changes in individual functioning (in the small to large range). The bibliotherapy condition performed better than expected with regard to pre‐post within‐group effect sizes on relationship functioning (small to medium effect sizes) and individual function (small effect sizes approaching medium).

Taken together, the findings consistently point in the same direction as previous large‐scale, nationwide US‐based studies on OR conducted by program developers (Doss et al. 2016, 2020). Also in consistency with previous research, effects were maintained over 3 months (Doss et al. 2019). These findings should be confirmed in a fully powered RCT, but the current study result encourages the study assumption of OR as an efficacious intervention when examined across cultures and by an independent research group. The finding that the OR was significantly more effective in reducing relationship negatives and communication conflict as compared to bibliotherapy fits well with the different foci of these two types of self‐help; the OR focuses on identifying, understanding, and solving one or two core relationship problem(s), and the bibliotherapy focuses on reading and discussing positive and prosocial relationship behavior—including addressing issues of disagreement. Although this difference in foci seems to be the obvious interpretation, it is important to note that the study conditions differ on two other aspects: the format (online program vs. book) and the level of support (five coach calls vs. receiving a reading guide). Future research is needed to isolate the effect of each of the aspects on reductions in conflict and relationship negatives.

In overall terms, the within‐group effects across the OR and bibliotherapy conditions were similar rather than different. This was unexpected and called for some interpretation. The similarities could reflect effects such as shared study conditions. For instance, couples may improve spontaneously over time, from their own decision to take active steps toward improving their relationship or from study procedures such as filling in questionnaires while reflecting on the relationship. While the fact that we only detected significant between‐group differences in terms of communication conflict and relationship negatives could indicate weaker support for the program, similarities could also reflect the real effects of the bibliotherapy condition. We may detect an actual but understudied promise of bibliotherapy (in particular, in terms of improving relationship functioning). To the best of our knowledge, only three previous RCTs—beyond the current pilot—have included a bibliotherapy condition, and in all cases, bibliotherapy is used as an active control condition (Babcock et al. 2013; Barton et al. 2018; Halford, Sanders, and Behrens 2001). Comparing bibliotherapy to couple education across 4 years for both high‐ and low‐risk couples, Halford, Sanders and Behrens (2001) found that lower‐risk couples (in contrast to higher‐risk couples) fared better in the bibliotherapy condition on long‐term outcomes. These previous findings suggest that a self‐help book might be a good choice for low‐risk couples (Stewart et al. 2016), and the current findings add that a self‐help book focused on prosocial behavior might be as good a choice for improving positives (e.g., intimacy) whereas the OR program might be a better choice for improving negatives (e.g. arguments). Our preliminary results should be considered in light of a larger field of evidence showing that self‐help books are found to be effective tools for individual issues (such as depression and anxiety disorders, for a review see Norcross 2000). We lack solid testing of bibliotherapy with more reliable measures of completion data for different samples in general and for parents in conflict in particular. With the indications from the current study, more research on the potential of bibliotherapy for different types of populations and couple issues seems relevant. Such future research could, for instance, include studies where the bibliotherapy group also receives coach calls, as we cannot rule out that some of the between‐group differences were due to the OR couples being more engaged as a result of receiving coach calls. There seems to be a potential utility of the bibliotherapy option, not to replace the OR option, but as a second choice of less intensive care.

A limitation of the current study is that the design does not enable us to isolate the effect of unaided, spontaneous remission of symptoms over time. Two recent studies, in which waitlisted couples were waiting for online interventions, found that although the majority of couples remained relationally distressed, there were significant improvements over three (Doss et al. 2016) or 6 months of waiting (Barton et al. 2021). As the authors of these studies discuss, such findings may indicate that online interventions, compared to studies on face‐to‐face therapy, reach couples at an earlier and more dynamic stage of relationship deterioration during which unaided, spontaneous remission is possible. Whereas previous literature on behavioral couple therapy concluded that there is no need for waitlists because distressed couples do not improve by themselves (Baucom, Hahlweg, and Kuschel 2003), a conclusion supported also in recent meta‐analyses (Roddy et al. 2020), our study results call for a full‐scale study with a waitlist condition in addition to the active control to control for unaided, spontaneous remission of symptoms, as this may account for parts of the within‐group changes in the current study.

A second limitation is that the current study relied on a small sample size lacking statistical power to detect significant small‐medium between‐group effects, increasing the possibility of a type II error. However, while it is encouraging that the differences are in the expected direction, we cannot rule out that the smaller‐sized differences between the two groups were an artifact of random error. Also, while the within‐group changes are encouraging, we cannot conclude a treatment effect. Accordingly, it will be important to continue investigations of the OR in a Danish context in a larger sample with sufficient statistical power. Findings from this pilot contribute by informing such power calculations as well as providing a solid test of the needed study methods.

Among the important contributions of the current study is the test of implementation feasibility before a large‐scale RCT. During the last couple of decades, heavy investments of research money have facilitated hundreds of large‐scale RCTs on the effects of technology‐based interventions. Yet, the return on research investments may fail if real‐world organizations are not willing to embrace these treatments. For example, as concluded in the Danish Feasibility Report, to provide existing clients with an updated and secure software solution, the following organizational demands must be met: (1) at least one in‐house staff member with software knowledge, (2) a staffed helpline to support users with technical issues, (3) an ongoing collaboration with a company specialized in digital solutions for data security and larger updates, (4) a robust marketing strategy to ensure a large inflow of couples to a novel treatment, (5) a model for recruiting, training, and supervising a new and less specialized workforce (the coaches), (6) external funding to cover 4‐year platform updates, and (7) a strategy to handle the integration of technology‐based treatment into existing services and avoid potential disruption of existing services. These requirements underscore that the introduction of technology‐based treatments into a new clinical environment is a complex, collaborative effort that requires a well‐resourced organization with a willingness to make organizational changes, an ability to take the intervention to scale, and a long‐term dedication.

Nevertheless, the overall conclusion of the Danish Feasibility Report was that OR is a relevant, attractive, and tolerable intervention for the Center for Family Development as an organization as well as for the couples that the organization serves today. Moreover, the implementation of the OR would allow the Center to serve and help couples nationwide. Couples in the OR condition had very high rates of client satisfaction and completion, although not completely at the level of the original US benchmark study (Doss et al. 2016). Regarding time allocation, Danish coaches dedicated more time per couple than what was noted in the benchmark study. However, even with the time‐use adjustments considered, a self‐sustaining pricing model appeared feasible, provided there is a minimum annual influx of 150 paying couples. This pricing strategy assumes that the platform is pre‐existing and operational. To cover the expenses of constructing or significantly updating the platform, a considerably larger number of paying couples or the acquisition of external funding would be necessary. Overall, implementing the OR not only enables but also necessitates a strategy for achieving large‐scale reach.

In conclusion, this pilot randomized controlled trial highlights the replicability of previous positive findings on the OR and sheds light on the overall potential of self‐help options to alleviate couple conflict and distress in parenting couples. The OR showed particular promise in reducing couple conflict and relationship negatives, an important asset of intervention offered in the context of parenting couples. Feasibility results were positive for both couples (i.e., couple evaluation, completion) and the organization (i.e., technical, organizational, and economical). However, a local implementation of the OR program requires a resourceful organization, includes organizational change, and necessitates a strategy for going to scale. We believe that successful replication of the findings in Denmark provides stronger evidence that the OR program is effective across different cultural contexts and extends the generalizability of the program's effectiveness beyond the original US setting.

It is possible that non‐tech self‐help options have suffered some oversight due to the rapid development and commercial potentials of technology‐based interventions. This study underscores the importance of not losing sight of low‐tech options at hand, which are readily available in all local settings and impose no adaptation challenges for clients, clinicians, or organizations. Both high‐tech solutions like the OR and non‐tech alternatives such as bibliotherapy may prove to be essential parts of building a coherent system of support that matches the needs and preferences of parenting couples.

## Supporting information

Supporting information.

## Endnotes

1 Furthermore, an RCT and a randomized trial have been conducted with a focus on the level of coach support in the OR (Roddy, Rothman, and Doss 2018; Rothman, Roddy, and Doss 2019). However, as the current study does not test varying levels of coach support, we focus less on this part of the existing literature.

2 Doss et al. (2016) found medium to large between‐group effect sizes on individual functioning for a subsample of individuals who began the program reporting problematic individual functioning. However, the within‐group effect sizes were not presented for this subsample of individuals.

## References

[jmft12762-bib-0001] Babcock, J. C. , J. M. Gottman , K. D. Ryan , and J. S. Gottman . 2013. “A Component Analysis of a Brief Psycho‐Educational Couples’ Workshop: One‐Year Follow‐Up Results.” Journal of Family Therapy 35, no. 3: 252–280. 10.1111/1467-6427.12017.

[jmft12762-bib-0002] Barton, A. W. , S. R. H. Beach , A. C. Wells , et al. 2018. “The Protecting Strong African American Families Program: A Randomized Controlled Trial With Rural African American Couples.” Prevention Science 19, no. 7: 904–913. 10.1007/s11121-018-0895-4. 29629507 PMC6177321

[jmft12762-bib-0003] Barton, A. W. , J. A. Lavner , M. J. Hawrilenko , and B. D. Doss . 2021. “Trajectories of Relationship and Individual Functioning Among Waitlisted Couples for an Online Relationship Intervention.” Family Process 60, no. 4: 1233–1248. 10.1111/famp.12691. 34250609 PMC11229881

[jmft12762-bib-0004] Baucom, D. H. , K. Hahlweg , and A. Kuschel . 2003. “Are Waiting‐List Control Groups Needed in Future Marital Therapy Outcome Research?” Behavior Therapy 34, no. 2: 179–188. 10.1016/S0005-7894(03)80012-6.

[jmft12762-bib-0005] Carlbring, P. , G. Andersson , P. Cuijpers , H. Riper , and E. Hedman‐Lagerlöf . 2018. “Internet‐Based vs. Face‐to‐Face Cognitive Behavior Therapy for Psychiatric and Somatic Disorders: An Updated Systematic Review and Meta‐Analysis.” Cognitive Behaviour Therapy 47, no. 1: 1–18. 10.1080/16506073.2017.1401115. 29215315

[jmft12762-bib-0006] Center for Family Development . 2021. OurRelationship. Feasibility‐Rapport [ENG: OurRelationship. Feasibility Report]. Aarhus, Denmark: Center for Familieudvikling.

[jmft12762-bib-0009] Christensen, A. 2010. “A Unified Protocol for Couple Therapy.” In Enhancing Couples: The Shape of Couple Therapy to Come, edited by K. Hahlweg , M. Grawe‐Gerber , and D. H. Baucom , 33–46. Cambridge, MA: Hogrefe Publishing.

[jmft12762-bib-0007] Christensen, A. , B. D. Doss , and N. S. Jacobson . 2014. Reconcilable Differences: Rebuild Your Relationship by Rediscovering the Partner You Love‐‐Without Losing Yourself, 2nd ed. New York: The Guilford Press.

[jmft12762-bib-0008] Christensen, A. , B. D. Doss , and N. S. Jacobson . 2020. Integrative Behavioral Couple Therapy: A Therapist's Guide to Creating Acceptance and Change, Second Edition. New York: W. W. Norton & Company.

[jmft12762-bib-0010] Cohen, S. , T. Kamarck , and R. Mermelstein . 1983. “A Global Measure of Perceived Stress.” Journal of Health and Social Behavior 24, no. 4: 385–396. 10.2307/2136404. 6668417

[jmft12762-bib-0011] Cordova, J. V. 2009. The Marriage Checkup: A Scientific Program for Sustaining and Strengthening Marital Health. Lanham, MD: Jason Aronson.

[jmft12762-bib-0012] Cordova, J. V. , C. J. E. Fleming , M. I. Morrill , et al. 2014. “The Marriage Checkup: A Randomized Controlled Trial of Annual Relationship Health Checkups.” Journal of Consulting and Clinical Psychology 82, no. 4: 592–604. 10.1037/a0037097. 24932565 PMC4115001

[jmft12762-bib-0013] Cummings, E. M. , and P. T. Davies . 2002. “Effects of Marital Conflict on Children: Recent Advances and Emerging Themes in Process‐Oriented Research.” Journal of Child Psychology and Psychiatry 43, no. 1: 31–63. 10.1111/1469-7610.00003. 11848336

[jmft12762-bib-0014] Debrot, A. , D. Schoebi , M. Perrez , and A. B. Horn . 2013. “Touch as an Interpersonal Emotion Regulation Process in Couples’ Daily Lives: The Mediating Role of Psychological Intimacy.” Personality and Social Psychology Bulletin 39, no. 10: 1373–1385. 10.1177/0146167213497592. 23885034

[jmft12762-bib-0015] Doig, G. S. , and F. Simpson . 2005. “Randomization and Allocation Concealment: A Practical Guide for Researchers.” Journal of Critical Care 20, no. 2: 187–191. 10.1016/j.jcrc.2005.04.005. 16139163

[jmft12762-bib-0016] Doss, B. D. , L. A. Benson , E. J. Georgia , and A. Christensen . 2013. “Translation of Integrative Behavioral Couple Therapy to a Web‐Based Intervention.” Family Process 52, no. 1: 139–153. 10.1111/famp.12020. 25408094 PMC4239659

[jmft12762-bib-0017] Doss, B. D. , L. N. Cicila , E. J. Georgia , et al. 2016. “A Randomized Controlled Trial of the Web‐Based OurRelationship Program: Effects on Relationship and Individual Functioning.” Journal of Consulting and Clinical Psychology 84, no. 4: 285–296. 10.1037/ccp0000063. 26999504 PMC4804631

[jmft12762-bib-0018] Doss, B. D. , L. K. Feinberg , K. Rothman , M. K. Roddy , and J. S. Comer . 2017. “Using Technology to Enhance and Expand Interventions for Couples and Families: Conceptual and Methodological Considerations.” Journal of Family Psychology 31, no. 8: 983–993. 10.1037/fam0000349. 29309184 PMC5761076

[jmft12762-bib-0019] Doss, B. D. , K. Knopp , M. K. Roddy , K. Rothman , S. G. Hatch , and G. K. Rhoades . 2020. “Online Programs Improve Relationship Functioning for Distressed Low‐Income Couples: Results From a Nationwide Randomized Controlled Trial.” Journal of Consulting and Clinical Psychology 88, no. 4: 283–294. 10.1037/ccp0000479. 32134290

[jmft12762-bib-0020] Doss, B. D. , M. K. Roddy , M. M. Llabre , E. Georgia Salivar , and A. Jensen‐Doss . 2020. “Improvements in Coparenting Conflict and Child Adjustment Following an Online Program for Relationship Distress.” Journal of Family Psychology 34, no. 1: 68–78. 10.1037/fam0000582. 31380688 PMC6994328

[jmft12762-bib-0021] Doss, B. D. , M. K. Roddy , K. M. Nowlan , K. Rothman , and A. Christensen . 2019. “Maintenance of Gains in Relationship and Individual Functioning Following the Online OurRelationship Program.” Behavior Therapy 50, no. 1: 73–86. 10.1016/j.beth.2018.03.011. 30661568 PMC6347396

[jmft12762-bib-0022] Eurostat . 2021. Eurostat: Divorce Indicators. https://ec.europa.eu/eurostat/databrowser/view/demo_ndivind/default/bar?lang=en.

[jmft12762-bib-0023] Fincham, F. D. , and R. Rogge . 2010. “Understanding Relationship Quality: Theoretical Challenges and New Tools for Assessment.” Journal of Family Theory & Review 2, no. 4: 227–242. 10.1111/j.1756-2589.2010.00059.x.

[jmft12762-bib-0024] Funk, J. L. , and R. D. Rogge . 2007. “Testing the Ruler With Item Response Theory: Increasing Precision of Measurement for Relationship Satisfaction With the Couples Satisfaction Index.” Journal of Family Psychology 21, no. 4: 572–583. 10.1037/0893-3200.21.4.572. 18179329

[jmft12762-bib-0025] Georgia Salivar, E. , K. Knopp , M. K. Roddy , L. A. Morland , and B. D. Doss . 2020. “Effectiveness of Online Ourrelationship and ePREP Programs for Low‐Income Military Couples.” Journal of Consulting and Clinical Psychology 88, no. 10: 899–906. 10.1037/ccp0000606. 33048570

[jmft12762-bib-0026] Georgia Salivar, E. J. , M. K. Roddy , K. M. Nowlan , and B. D. Doss . 2018. “Effectiveness of the Online Ourrelationship Program for Underserved Couples.” Couple and Family Psychology: Research and Practice 7, no. 3–4: 212–226. 10.1037/cfp0000110.

[jmft12762-bib-0027] Georgia Salivar, E. J. , K. Rothman , M. K. Roddy , and B. D. Doss . 2020. “Relative Cost Effectiveness of In‐Person and Internet Interventions for Relationship Distress.” Family Process 59, no. 1: 66–80. 10.1111/famp.12414. 30537091

[jmft12762-bib-0028] Gottman, J. M. , and N. Silver . 1999. The Seven Principles For Making Marriage Work: A Practical Guide From the Country's Foremost Relationship Expert. Harmony Books.

[jmft12762-bib-0029] Halford, W. K. and Markman, H. J. , ed. 1997. Clinical Handbook of Marriage and Couples Interventions. New York: Wiley.

[jmft12762-bib-0030] Halford, W. K. , M. R. Sanders , and B. C. Behrens . 2001. “Can Skills Training Prevent Relationship Problems in At‐Risk Couples? Four‐Year Effects of a Behavioral Relationship Education Program.” Journal of Family Psychology 15, no. 4: 750–768. 10.1037//0893-3200.15.4.750. 11770479

[jmft12762-bib-0031] Hatch, S. G. , K. Knopp , Y. Le , et al. 2022. “Online Relationship Education for Help‐Seeking Low‐Income Couples: A Bayesian Replication and Extension of the OurRelationship and ePREP Programs.” Family Process 61, no. 3: 1045–1061. 10.1111/famp.12710. 34383314

[jmft12762-bib-0032] Hedman‐Lagerlöf, E. , P. Carlbring , F. Svärdman , H. Riper , P. Cuijpers , and G. Andersson . 2023. “Therapist‐Supported Internet‐Based Cognitive Behaviour Therapy Yields Similar Effects as Face‐to‐Face Therapy for Psychiatric and Somatic Disorders: An Updated Systematic Review and Meta‐Analysis.” World Psychiatry 22, no. 2: 305–314. 10.1002/wps.21088. 37159350 PMC10168168

[jmft12762-bib-0033] Jacobson, N. S. , and A. Christensen . 1998. Acceptance and Change in Couple Therapy: A Therapist's Guide to Transforming Relationships. New York: W.W. Norton & Company.

[jmft12762-bib-0034] Lyubomirsky, S. 2007. The How of Happiness: A New Approach to Getting the Life You Want. New York: Penguin Press.

[jmft12762-bib-0035] McNeish, D. , L. M. Stapleton , and R. D. Silverman . 2017. “On the Unnecessary Ubiquity of Hierarchical Linear Modeling.” Psychological Methods 22, no. 1: 114–140. 10.1037/met0000078. 27149401

[jmft12762-bib-0036] Mitchell, E. A. , Y. Le , S. G. Hatch , S. Guttman , and B. D. Doss . 2023. “Effects of Online Relationship Programs for Low‐Income Couples During the Perinatal Period.” Behaviour Research and Therapy 167: 104337. 10.1016/j.brat.2023.104337. 37327534

[jmft12762-bib-0037] Nguyen, T. D. , C. C. Attkisson , and B. L. Stegner . 1983. “Assessment of Patient Satisfaction: Development and Refinement of a Service Evaluation Questionnaire.” Evaluation and Program Planning 6, no. 3–4: 299–313. 10.1016/0149-7189(83)90010-1. 10267258

[jmft12762-bib-0038] Norcross, J. C. 2000. “Here Comes the Self‐Help Revolution in Mental Heath.” Psychotherapy: Theory, Research, Practice, Training 37, no. 4: 370–377. 10.1037/0033-3204.37.4.370.

[jmft12762-bib-0039] Olsen, L. R. , D. V. Jensen , V. Noerholm , K. Martiny , and P. Bech . 2003. “The Internal and External Validity of the Major Depression Inventory in Measuring Severity of Depressive States.” Psychological Medicine 33, no. 2: 351–356. 10.1017/S0033291702006724. 12622314

[jmft12762-bib-0040] Osman, A. , C. L. Bagge , P. M. Gutierrez , L. C. Konick , B. A. Kopper , and F. X. Barrios . 2001. “The Suicidal Behaviors Questionnaire‐Revised (SBQ‐R): Validation With Clinical and Nonclinical Samples.” Assessment 8, no. 4: 443–454. 10.1177/107319110100800409. 11785588

[jmft12762-bib-0041] Politiken . 2019. Flere kommuner tilbyder gratis parterapi. https://politiken.dk/danmark/forbrug/art7295957/Flere-kommuner-tilbyder-gratis-parterapi.

[jmft12762-bib-0042] Rhoades, G. K. , S. M. Stanley , and H. J. Markman . 2009. “Couples’ Reasons for Cohabitation: Associations With Individual Well‐Being and Relationship Quality.” Journal of Family Issues 30, no. 2: 233–258. 10.1177/0192513X08324388. 19756225 PMC2743430

[jmft12762-bib-0043] Robles, T. F. , R. B. Slatcher , J. M. Trombello , and M. M. McGinn . 2014. “Marital Quality and Health: A Meta‐Analytic Review.” Psychological Bulletin 140, no. 1: 140–187. 10.1037/a0031859. 23527470 PMC3872512

[jmft12762-bib-0044] Roddy, M. K. , K. Knopp , E. Georgia Salivar , and B. D. Doss . 2021. “Maintenance of Relationship and Individual Functioning Gains Following Online Relationship Programs for Low‐Income Couples.” Family Process 60, no. 1: 102–118. 10.1111/famp.12541. 32352573

[jmft12762-bib-0045] Roddy, M. K. , G. K. Rhoades , and B. D. Doss . 2020. “Effects of ePREP and OurRelationship on Low‐Income Couples’ Mental Health and Health Behaviors: A Randomized Controlled Trial.” Prevention Science 21, no. 6: 861–871. 10.1007/s11121-020-01100-y. 32108304

[jmft12762-bib-0046] Roddy, M. K. , K. Rothman , and B. D. Doss . 2018. “A Randomized Controlled Trial of Different Levels of Coach Support in an Online Intervention for Relationship Distress.” Behaviour Research and Therapy 110: 47–54. 10.1016/j.brat.2018.09.002. 30240874

[jmft12762-bib-0047] Roddy, M. K. , L. M. Walsh , K. Rothman , S. G. Hatch , and B. D. Doss . 2020. “Meta‐Analysis of Couple Therapy: Effects Across Outcomes, Designs, Timeframes, and Other Moderators.” Journal of Consulting and Clinical Psychology 88, no. 7: 583–596. 10.1037/ccp0000514. 32551734

[jmft12762-bib-0048] Rothman, K. , M. K. Roddy , and B. D. Doss . 2019. “Completion of a Stand‐Alone Versus Coach‐Supported Trial of a Web‐Based Program for Distressed Relationships.” Family Relations 68, no. 4: 375–389. 10.1111/fare.12378.

[jmft12762-bib-0049] Simpson, L. E. , D. C. Atkins , K. S. Gattis , and A. Christensen . 2008. “Low‐Level Relationship Aggression and Couple Therapy Outcomes.” Journal of Family Psychology 22, no. 1: 102–111. 10.1037/0893-3200.22.1.102. 18266537

[jmft12762-bib-0050] Slatcher, R. B. , and E. Selcuk . 2017. “A Social Psychological Perspective on the Links Between Close Relationships and Health.” Current Directions in Psychological Science 26, no. 1: 16–21. 10.1177/0963721416667444. 28367003 PMC5373007

[jmft12762-bib-0051] Spencer, C. M. , S. M. Stith , and B. Cafferky . 2022. “What Puts Individuals at Risk for Physical Intimate Partner Violence Perpetration? A Meta‐Analysis Examining Risk Markers for Men and Women.” Trauma, Violence & Abuse 23, no. 1: 36–51. 10.1177/1524838020925776. 32431231

[jmft12762-bib-0052] Stanley, S. M. , S. L. Blumberg , and H. J. Markman . 1999. “Helping Couples Fight for Their Marriages: The PREP Approach.” In Preventive Approaches in Couples Therapy, edited by R. Berger and M. T. Hannah , 279–303. Philadelphia: Brunner/Mazel.

[jmft12762-bib-0053] Stewart, J. W. , K. Bradford , B. J. Higginbotham , and L. Skogrand . 2016. “Relationship Help‐Seeking: A Review of the Efficacy and Reach.” Marriage & Family Review 52, no. 8: 781–803. 10.1080/01494929.2016.1157559.

[jmft12762-bib-0054] Stith, S. M. , N. M. Green , D. B. Smith , and D. B. Ward . 2008. “Marital Satisfaction and Marital Discord as Risk Markers for Intimate Partner Violence: A Meta‐Analytic Review.” Journal of Family Violence 23, no. 3: 149–160. 10.1007/s10896-007-9137-4.

[jmft12762-bib-0055] Stølen Due, M. 2016. Pas på parforholdet, når kærligheden er kommet for at blive [ENG: Take Care of Your Relationship—When Love has Come to Stay]. Copenhagen: Kristeligt Dagblads Forlag.

[jmft12762-bib-0056] Trillingsgaard, T. , H. N. Fentz , M. Hawrilenko , and J. V. Cordova . 2016. “A Randomized Controlled Trial of the Marriage Checkup Adapted for Private Practice.” Journal of Consulting and Clinical Psychology 84, no. 12: 1145–1152. 10.1037/ccp0000141. 27599227

[jmft12762-bib-0057] Trillingsgaard, T. , T. K. Sørensen , and H. N. Fentz . 2019. “Relationship Help‐Seeking in a Danish Population Sample.” Journal of Marital and Family Therapy 45, no. 3: 380–394. 10.1111/jmft.12347. 30084232

[jmft12762-bib-0058] Trillingsgaard, T. L. , H. N. Fentz , M. Simonsen , and R. E. Heyman . 2019. “The Prevalence of Intimate Partner Violence Among Couples Signing Up for Universally Offered Parent Preparation.” PLoS One 14, no. 10: e0223824. 10.1371/journal.pone.0223824. 31613936 PMC6793941

[jmft12762-bib-0059] WHOQOL Group . 1998. Programme on Mental Health, WHOQQL User Manual. www.who.int/publications/i/item/WHO-HIS-HSI-Rev.2012.03.

