# Supplementary material for: Comparing the OurRelationship Program and Bibliotherapy for Parents Experiencing Couple Distress: A Randomized Pilot
Source: J Marital Fam Ther. 2025 Jan 7;51(1):e12762. doi: 10.1111/jmft.12762 (PMC11707500; doi:10.1111/jmft.12762)
Supplement: Supplementary file 1 — Supporting information. [file JMFT-51-0-s001.pdf]

Supplementary Appendix

"Comparing the OurRelationship Program and Bibliotherapy for Parents Experiencing Couple Distress: A Randomized Pilot"

January 2025

Table S1. Baseline characteristics by experimental condition

	**Bibliotherapy**		**OurRelationship**		**Difference**
	M/%	*SD*	*M/%*	*SD*	
Age (mean years)	38.53	8.01	38.95	8.40	ns.
Relationship length (mean years)	14.21	9.75	11.75	5.64	ns.
Married*	47 %		86 %		$p < .05$
Completed high school	85 %		70 %		ns.
Previous experience with therapy	21 %		41 %		ns.
Currently employed	82 %		91 %		ns.

Note. N = 78 individuals, ns. = not significant at the 5-percent level ($p > .05$).

Table S2. Pre-defined feasibility of the OurRelationship in clinical practice

Feasibility test	Predefined criteria
1. *Liking*	We expect client satisfaction levels to be comparable to the original study (Doss et al., 2016), with a mean score of 26.81 (*SD* =4.44) on the eight-item Client-evaluation of Services Questionnaire. We will accept 0.3 *SD* lower client satisfaction compared to the original study
2. *Completion*	We expect an 80 % completion rate of *Observe* and *Understand* modules or more.
3. *Coaching*	Comparable to reports in Doss et al. (2016), we expect coaches on average to be able to spend an hour or less in total with each couple on the telephone over the study period and some additional time (max. 15 min. pr. couple) supporting couples with reminders and re-scheduling through a chat function. In the original RCT coaches received initial training in the intervention and weekly supervision.
4. *Technical setup*	We expect that the data hosting setup, legal protection, software skills of AU IT with some support from Kineo (US) will be sufficient for needs of running the program and supporting clients. We expect the Danish Data protection agency will approve of the setup.

5. Organizational setup	We expect that a real-world clinical organization providing face-to-face couple interventions, in this pilot project represented by Center for Familieudvikling Aarhus, will meet all organizational requirements of running the program except for IT technical requirements and IT support.
6. Price expectancy	We ask couples what they would be willing to pay for program participation outside research.
7. Costs	We calculate the cost per couple. This includes all costs related to the technical aspects (e.g., IT, hosting) and workforce (e.g., coaches, secretary). We compare both the cost per couple and the effect of OurRelationship program with that of PREP and the Marriage checkup ("Par-tjek") as offered in a current municipality setting in Denmark.
